# The neural mechanism of communication between graduate students and advisers in different adviser-advisee relationships

**DOI:** 10.1038/s41598-024-58308-z

**Published:** 2024-05-23

**Authors:** Yan Zhang, Peipei Wu, Simiao Xie, Yan Hou, Huifen Wu, Hui Shi

**Affiliations:** 1 School of Education, Huazhong University of Science and Technology, Wuhan, 430074 Hubei China; 2 School of Education, Hubei Engineering University, Xiaogan, 432100 Hubei China; 3 Mental Health Education Center, Jinan University, Guangzhou, 510631 Guangdong China; 4 Mental Health Education Center, Hubei University for Nationalities, Enshi, 450004 Hubei China; 5 Department of Clinical Psychology, Beijing Chao-Yang Hospital, Capital Medical University, Beijing, 100020 China; 6 Research Center for Innovative Education and Critical Thinking, Huazhong University of Science and Technology, Wuhan, 430074 Hubei China

**Keywords:** Adviser-advisee communication, Adviser-advisee relationships, FNIRS, Neuroscience, Health care

## Abstract

Communication is crucial in constructing the relationship between students and advisers, ultimately bridging interpersonal interactions. Only a few studies however explore the communication between postgraduate students and advisers. To fill the gaps in the empirical researches, this study uses functional near-infrared spectroscopy (FNIRS) techniques to explore the neurophysiology differences in brain activation of postgraduates with different adviser-advise relationships during simulated communication with their advisers. Results showed significant differences in the activation of the prefrontal cortex between high-quality and the low-quality students during simulating and when communicating with advisers, specifically in the Broca's areas, the frontal pole, and the orbitofrontal and dorsolateral prefrontal cortices. This further elucidated the complex cognitive process of communication between graduate students and advisers.

## Introduction

The adviser-advisee relationship is an integral part of the interaction between graduate students and advisers, and it has profound influences on many aspects of students’ academic life^1^. According to Morpurgo^2^, a qualified adviser-advisee relationship involves the following major factors: intensity of personal involvement, immediate consequences for classroom practice, stimulation and ego support by meaningful association and initiation by teacher rather than by outsider. Nadler^3^ provided further support for the notion that a positive adviser-advisee relationship are largely associated with advisers’ empathy and communication behavior-related items(i.e., reported frequency of visits to one's adviser, length of advising visits, quality of advising, frequency of discussing personal matters, and frequency of talking about personal, non-academic matters). The satisfying interactions between graduates and advisers can promote long-term positive relationships, and vice versa will produce negative ones. A positive adviser-advisee relationship is essential to establish a pleasant learning atmosphere with graduate students at school, while a terrible relationship might be a potential threat to students’ normal school life. Recent violent events in colleges and universities related to adviser-advisee relationship have brought more attention to this topic. There exits a growing body of research having indicated that as a kind of interpersonal relationship, adviser-advisee relationship is closely related to the psychological distance between the two sides. For example, according to Hui (and other scholars in psychology), adviser-advisee relationship is centered on the cognitive and emotional interaction between the adviser and the student, providing a feedback loop on each other's psychological links shaped by functional communication while “getting along^4^”. And for Liu Zhi, the stable establishment process of adviser-advisee relationship follows the benign interaction between advisers and postgraduates^5^, but the focus of interaction remains to be around advisers. Although the ideal adviser-advisee relationship is complex and multi-dimensional^6^, communication is an important channel through which this relationship can be connected.

Much of the evidence documenting a link between adviser-advisee relationship and communication has come from investigation studies. For instance, Wu and Han chose master advisers as the interview object to conduct open and in-depth interviews on factors affecting the adviser-advisee relationship. Five important influencing factors were abstracted from the original interview data, with communication being one of them^7^. Other researchers meanwhile surveyed 135 students and 24 teachers and found that 100% of teachers while 88% of students believed that communication in teacher-student relationship was pivotal: effective communication between teachers and students was an important factor in achieving high-quality education^8^. To some extend, communication also affects students’ academic performance. Katz and Hartnett distinguished the important factors perceived by graduate students in their academic education and found that the communication between graduate students and their advisers was likely the most significant factor^9^. Mccuen investigated more than 100 postgraduate students and found that most students agree on adviser-advisee’s communication and its profound impact on their academic research^10^.

There exists a close relationship between adviser-advisee relationship and communication. Wang’s research found that the poor communication between postgraduate students and advisers may be related to the variation of teacher-student relationship^11^. Mainhard combined the concept of interpersonal relationship management behavior mode and confirmed that the harmonious and positive adviser-advisee relationship effectively promotes the academic success of postgraduate students. Simultaneously, the study also found the influence of interpersonal communication mode on the construction of an enriching adviser-advisee relationship^12^. Although these studies have focused on exploring the important role of communication in adviser-advisee relationship, due to the limitations of research methods, it is still not possible to reveal the interactive relationship between communication and adviser-advisee relationship more deeply.

As illustrated in the examples above, adviser-advisee communication is influenced by the adviser-advisee relationship, and it also plays an important role in the construction of the adviser-advisee relationship. However, research that directly explores the internal connection between communication and guidance through experimental methods is still limited. In particular, there remains no clear and direct neurophysiological evidence to confirm the relationship between teacher-student communication and adviser-advisee relationship. Here we take a first step to delineate the neural processes involved in students’ imaginary communication with advisors by using the realistic presented problem paradigm in combination with functional near-infrared spectroscopy (fNIRS), and special attention was paid to the role of different adviser-advisee relationships in this process. The reason why we used fNIRS technology is twofold. Firstly, functional near-infrared spectroscopy(fNIRS) has been widely used to measure prefrontal hemodynamic responses and it has been cross-validated with fMRI^13^. It is non-invasive, silent, low-cost, and it provides superior temporal resolution than fMRI and superior spatial resolution than electroencephalograpy^14^. Additionally, comparing to fMRI and EEG/MEG, fNIRS is more suitable to measure neural dynamics in social interaction context because the signal measured with fNIRS is not strongly influenced by the movement of participants. This special advantage of fNIRS can create experimental paradigms that resemble real-life situations more closely than classic studies^15^. Therefore, using fNIRS to pinpoint the neurocognitive alterations that occur specifically during attempts to establish an effective communication could ultimately improve our understanding of students’ brain systems associated with cognition, social interaction under different adviser-advisee relationships, and may provide a reference for exploring the teaching effect in different teacher-student relationship and evaluating the efficacy of future education interventions.

At present, many studies have revealed the characteristics of the activity of neural mechanisms in the brain areas during communication. For example, when thinking about how to establish a conversation with advisor about solving a certain dilemma (the thinking stage), brain areas associated with speech production (e.g., Broca’s area) would be activated. It has been suggested that left posterior inferior frontal cortex (Broca’s area) is involved in language function such as language production^16^. A circumscribed damage to Broca’s area could result in temporary speech production difficulties^17^. What’s more, language production and processing is thought to require cognitive control, thus prefrontal cortex areas relating to cognitive processes such as problem-solving and working-memory should also be taken into consideration. When trying to planning and executing a conversation, the dorsolateral prefrontal cortex is an integral part of this process due to its special role in executive control network and the functional connectivity between linguistic networks and other functional networks, including corticocortical and subcortical circuits^18^. The orbitofrontal cortex is also thought to be connected with language-related areas in the inferior frontal gyrus^19^. Taken together, these findings suggest that the brain regions associated with language production and executive function require to be highly valued.

In the real teaching situations, for those students who do not have a good adviser-advisee relationship, they will naturally have an avoidance mentality when communicating with the advisor. Students need to overcome an unaccustomed and evasive mentality to organize the language of communication with the advisor and may have tension and discomfort themselves. They also need to pay more cognitive control and effort to successfully proceed the communication. Therefore, we hypothesized that during the thinking phase, compared with the group with good adviser-advisee relationship, the group with positive relationship would have higher activation in the brain regions related to language organization and cognitive control. While in the simulated communication stage, the group with high relationship was better able to organize a rigorous dialogue, the conversation process was relatively more pleasant, and the students were more involved. Therefore, in the communication phase, we speculate that the group with a better relationship would have a higher activation of brain areas related to cognitive control (for example, the DLPFC and the OFC).

This study therefore combines experimental paradigms and brain neuroscience research methods to verify the relationship between both and deepens the mutual influence mechanism of adviser-advisee communication and the learning guidance relationship to the brain nerve level. This allows for a more comprehensive understanding of the cognitive characteristics and neural mechanism of the pros and cons of learning guidance relationship in the communication process.

## Method

### Participants

To measure the quality of adviser-advisee relationships, we adopted the the five-item scale developed by Allen et al^20^*.* for it included the items of the main factors for establishing a positive or negative adviser-advisee relationship such as perceived benefits, relationship satisfaction and relational depth, which are also the important components of real-life mentoring practice. Students would consider their relationships with advisers much positive when they found their interactions effective, satisfied and high-quality, and the benefits they got from the relationships are mutual for both the adviser and the advisee. If they do not meet these criteria, they tend to establish a negative and less quality relationship. Following the scale, subjects with two dimensions of different adviser-advisee relationships were selected. Through the questionnaire survey, 160 questionnaires were obtained for postgraduate students in H University. The scores from the total 160 questionnaires were ranked from high to low, and in order to exclude the influence of the length of work experience with advisers on the results of this study, we matched the length of work experience for the two groups of positive and negative relationships. Thus, those who scored in the top 17% were selected as the group with positive adviser-advisee relationship (M = 15.85, SD = 2.196), while those who scored in the bottom 17% were selected as the group with negative adviser-advisee relationship (M = 9.67, SD = 2.201; t = 10.336, *p* = 0.000 < 0.001). There were 27 people in each group and a total of 54 participates, and there was no significant difference in the length of work experience with advisers between two groups (*p* = 0.211). There were 20 first-year graduates, 21 s-year graduates and 13 third-year graduates. The average age of the sample set reached 23.81 ± 2.155 years. Both groups had normal hearing, normal or corrected visual acuity, no visual impairment (such as color blindness or color weakness) and were right-handed. Originally 54 participants were conducted the experiment, but 12 people had to be excluded due to high motion or poor signal. We matched the numbers of people and the length of work experience with advisers in two groups. Ultimately there were 21 participants in the group with positive adviser-advisee relationship (M = 15.86, SD = 2.032), 21 participants in the group with negative adviser-advisee relationship (M = 9.33, SD = 2.373; t = 9.568, *p* = 0.000 < 0.001), and a total of 42 data was analyzed with no significant difference in the length of work experience with advisers between two groups (*p* = 0.268).There were 14 male and 28 female, 14 first-year graduates, 16 s-year graduates and 12 third-year graduates. The average age of the sample set reached 24.00 ± 2.130 years.

This study was performed in accordance Declaration of Helsinki and was approved by the Ethics Committee of School of Huazhong University of Science and Technology (IRB No. 20201004). Prior to the experiment, participants were clearly informed about the experimental content and the specific information of the instrument used. After agreeing on the experimental process and confirming that the subjects had no further questions, written informed consent was signed, and participants were given certain remuneration after the experiment. Experimental protocol was approved by the ethics committee of the affiliated school.

### Instruments and procedure

#### Realistic presented problem

The experiment task was adapted from the realistic presented problem (RPP) paradigm, which required individuals to solve an open, realistic problem in a novel way^21–23^. This experiment also made reference to the situational simulation task, and adapted the content of the pre-interviewed and assessed dilemma situation contents into a communication simulation task under the problem solving scenario. The situational materials provided in the RPP were rated by a total of 20 postgraduates based on a 7-point Likert scale. The specific assessment contents include: familiarity, which refers to the degree of familiarity with the scene described in the text; Frequency of the situation refers to the frequency of text description situation or similar situation that may occur in real learning life; The degree of difficulty, that is, the degree to which you would feel difficult or intractable if you were in the situation described by the text; Degree of control is the extent to which the situation in a written description is something you can solve or make better. All the difficult situations and their assessments are shown in Table 1. Situational simulation effectively reflects the habitual behaviors of individuals and shows their inherent thinking patterns and neural activities^24^. Therefore, the study explored the existence of any differences in cognitive neural level between students with different adviser-advisee relationships in the communication process with their advisers by simulating the plot of communication between graduate students and their advisers.

**Table 1:** Evaluation results of experimental materials.

Difficult situations	Familiarity	Frequency	Degree of difficulty	Degree of control
The time of graduation proposal is getting closer and closer, but you have no idea of your graduation thesis	4.32	3.68	5.95	3.74
Again and again, you try the scientific software that must be used during the research, still always meet the error	4.37	4.37	5.53	4.26
You want to revise your course paper for submission, but you can’t find the right journal	4.09	3.42	4.47	4.47
You have a topic you want to study, but your adviser will only allow you to study what she or he has specified	4.53	3.26	4.95	3.11
You have no idea where you want to go beyond course work and scientific research	4.42	3.63	4.68	4.11
Your adviser criticizes your attitude at the group meeting for not reaching out to him or her	4.32	2.89	5.00	4.05
You feel lost and anxious about your job and wonder where the future leads	4.94	4.68	5.84	3.21
There are too many study groups and you feel like you can’t handle too much collaborative learning	4.13	3.32	4.42	4.26
You and your adviser have completely different ideas about the design direction of your graduation thesis	5.26	3.00	5.47	3.21
You don’t think you want to do academic research, but your adviser has high hopes for your research	4.29	3.26	5.11	3.05
Because your adviser has criticized you in public, you feel nervous every time you face your adviser alone	4.16	2.58	5.26	3.05

In this task, the participants were first presented with the problem situations (see Table 1) encountered in the study and life of postgraduate students, and asked to think about this scenario: how to communicate with the adviser to seek his or her help to solve the current dilemma, that is, imagine how to ask for the adviser's help and how to solve the problem with his or her help. After the thinking stage, the participants were asked to imagine the adviser in front of them and simulate the communication process with him or her in person.

**Figure 1:**
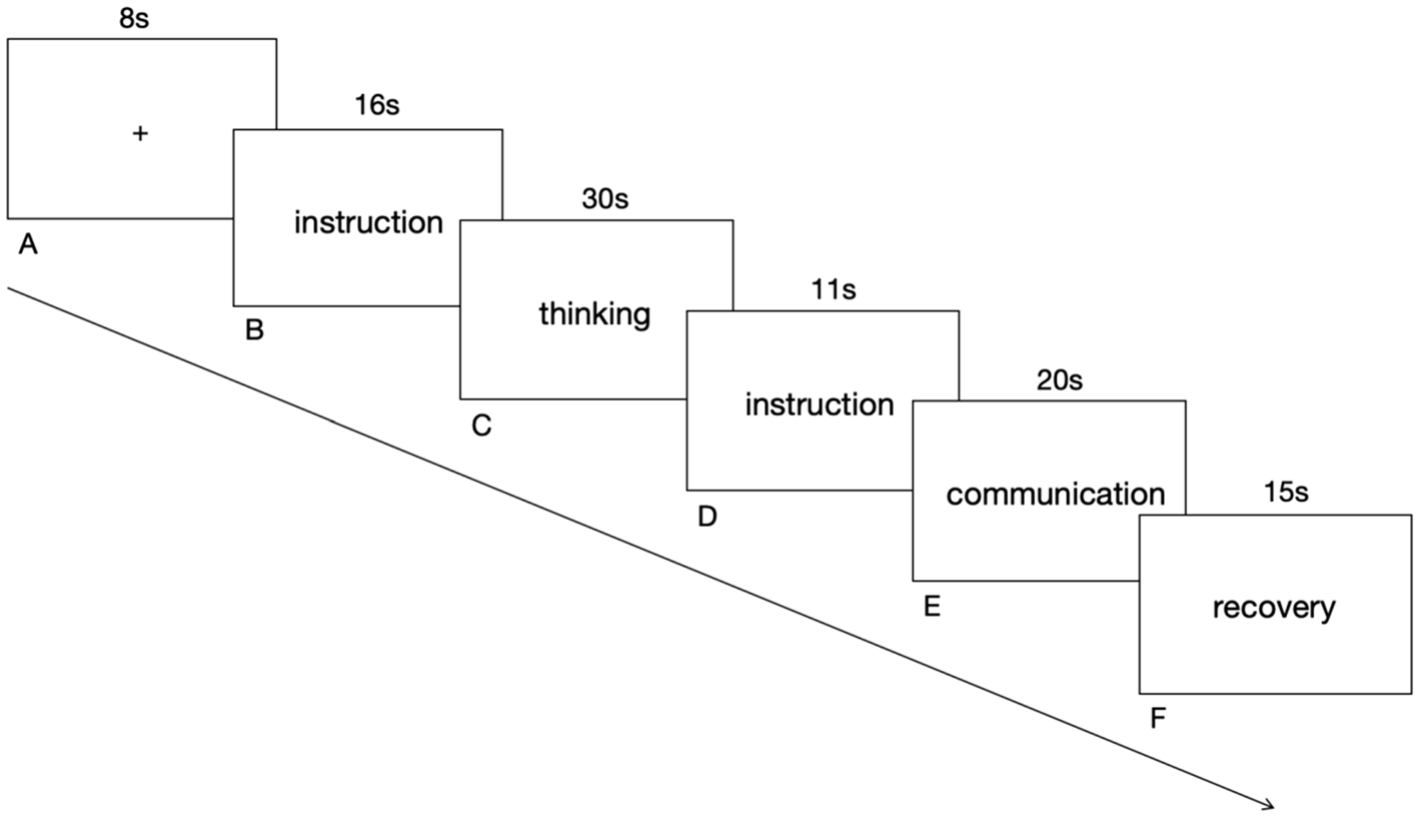
Schematic diagram of a single trial of the experimental flow. (**A**), Subjects begin by seeing an 8s fixation cue. (**B**), Sixteen-second instruction period in which subjects are presented with information regarding the difficult situation for that trial, and are informed of the incoming thinking period. (**C**), Thirty-second black screen for thinking (including 1 s buffering period). (**D**), Subjects are shown an eleven-second information that informs them to be ready to communicate with their advisers about the different situation. (**E**), Twenty-second black screen for communicating with the adviser. (**F**), Subjects have a 15 s recovery time to relax.

The entire task was presented on screen using the E-Prime 2.0 software (Psychology Software Tools, 2002). As shown in Fig. 1, all trails began with a 8-s fixation cross, following a 15-s instruction period in which subjects were presented with information regarding the difficult situations and the instructions to think about how to ask the adviser’s help about the scenario. At the end of the instruction is the thinking phase, when the computer screen will appear black for 30 s to let the participants think how to communicate with the advisor. A 1s empty screen would be displayed between the instruction phase and the thinking phase to buffer the visual aftereffect brought by the instructions, so that the participants can better immerse themselves in the situation. After thinking, subjects were then shown the following 11-s instruction: “After the black screen ends, please imagine your adviser in front of you. Please give a verbal report to simulate the scenario of on-site communication with your adviser, with a communication time of 20 s”. After that, the subjects will be presented with a black screen again for 20 s to orally simulate the communication process with the adviser. According to the oral reports of the subjects, the experimenter would judge the participants’ involvement in task completion, so as to screen out the participants who were not immersed in the simulation and communication with the adviser. After completing the task, the participants were given 15 s to relax. The whole experiment consisted of 1 practice trail and 10 formal trails, each of which lasted for 100 s, including the task of thinking about the situation and the task of simulating communication. The 10 formal trails were presented in a random order.

#### Functional near-infrared spectroscopy detection

NIRScout, a table top near-infrared brain imaging device developed by Shenzhen Hanxiang Biomedical Electronics Co., LTD., was used to detect the changes of oxygenated hemoglobin (HbO) in the prefrontal area of the subjects during the simulated communication task. The sampling frequency was 7.8125 Hz. Review the existing literature^25–27^, the prefrontal cortex is the main activated brain region for interpersonal communication, social interaction and communication, and other higher cognitive functions. Therefore, the prefrontal cortex is regarded as the area of interest in this study.

As has been mentioned in the introduction section, the Dorsolateral prefrontal cortex, Broca's area as well as the Orbitofrontal area are highly associated with language production and cognitive control. Therefore, in order to better explore the activities of different brain regions, we set the following three ROIs: Region 1 (CHs 2, 3, 4, 8, 9, 10, 17, 20) included all the channels that covered an area of 60% or more of the Dorsolateral prefrontal cortex. Region 2 (CHs1, 18) included all the channels that covered an area of 60% or more of Broca's area. Region 3 (CHs 4, 6, 11, 13, 16, 19) included all the channels that covered an area of 60% or more of the Orbitofrontal area.

The experiment adopted the design of 2 (Type of subjects: positive adviser-advisee relationship; negative adviser-advisee relationship) × 2 (Situation: thinking and preparation; Simulated communication), with the subject type as the inter-subject variable and the experiment situation as the within-subject variable. The dependent variable is the oxygenated hemoglobin value (HbO) obtained under different experimental conditions. To correct for multiple comparisons, we adopted a false-discovery rate (FDR) correction with q < 0.05^28^ in three regions respectively.

### Informed consent

Informed consent was obtained from all subjects involved in the study.

## Results

### Results of ANOVA of oxygenated hemoglobin value

The descriptive statistical analysis of oxygenated hemoglobin value of each channel in the two groups with positive and negative adviser-advisee relationships under different conditions was as follows: the values of activated oxygenated hemoglobin content in different groups of subjects detected by the device under different conditions were derived. After superposition and average, the values of different channels were entered into SPSS23.0 software for 2 (group: positive relationship/ negative relationship) × 2 (Situation: thinking/Simulated) repeated measures ANOVA to explore the main effect and interaction effect of the variables. Among them, only channels in Region 1 (CHs 2, 4) had significant interaction with the group oxygenated hemoglobin value, channel 18 had significant main effect margin of oxygenated hemoglobin value in different situations, and the other channels were not significant. The results are shown in Table 2.

**Table 2:** Descriptive analysis and the ANOVA results of HbO on the significant channels.

		Descriptive analysis results of HbO				HbO ANOVA results of the two groups					
		Group with negative relationship (n = 21)		Group with positive relationship (n = 21)		Variation	df	F	*p*	FDR q	η^2^
		M	SD	M	SD						
CH1	Thinking	1.788	7.807	− 2.831	7.955	Situation (A)	1	1.637	0.208		< 0.001
	Communication	− 0.060	13.028	5.063	14.173	Group (B)	1	0.010	0.920		0.039
						A × B	1	4.249*	0.046	0.092	0.096
CH2	Thinking	3.154	5.902	− 3.591	7.621	Situation (A)	1	0.687	0.412		0.017
	Communication	− 2.282	12.806	5.767	12.342	Group (B)	1	0.102	0.751		0.003
						A × B	1	9.718**	0.003	0.016	0.196
CH4	Thinking	3.251	5.687	0.012	2.867	Situation (A)	1	1.557	0.219		0.037
	Communication	− 3.140	7.191	2.689	5.763	Group (B)	1	2.007	0.157		0.049
						A × B	1	9.279**	0.004	0.016	0.188

Descriptive data are shown as mean *10,000, standard error *10,000; *,*p* < 0.05; **, *p* < 0.01;***,*p* < 0.001.

The results showed that in channel 1, the interaction between group and situation was significant (F (1,40) = 4.249, *p* = 0.046 < 0.05, η2 = 0.096). In channel 2, the interaction between group and context was significant (F (1,40) = 9.718, *p* = 0.003 < 0.01, η2 = 0.196). In channel 4, the interaction between group and context was significant (F (1,40) = 9.279, *p* = 0.004 < 0.01, η2 = 0.188).

In view of the significant interaction between group and situation in channel 1, further simple effect analysis showed that there was no significant difference in the groups of different adviser-advisee relationships in the simulated communication situation (*p* = 0.230), but there was significant difference in the edge of group difference in thinking situation (F (1, 40) = 3.680, *p* = 0.065, η2 = 0.083). The mean value of oxygenated hemoglobin in the group with negative adviser-advisee relationship was higher than that in the group with positive adviser-advisee relationship. In the negative group, the situation difference was not significant (*p* = 0.583). In the positive group, the situation difference was significant (F (1,40) = 5.580, *p* = 0.023 < 0.05, η2 = 0.122). The mean oxygenated hemoglobin content in the simulated communication situation was significantly higher than that in the thinking situation.

In view of the significant interaction between group and situation in channel 2, further simple effect test showed that there was significant difference in the quality of adviser-advisee relationship between the two groups in the thinking situation group (F(1,40) = 4.300, *p* = 0.045 < 0.05, η2 = 0.097), the mean value of oxygenated hemoglobin content in the negative group was significantly lower than that in the positive group. There was also significant difference in the thinking situation group (F (1,40) = 10.2846, *p* = 0.003 < 0.01,. η2 = 0.205), the mean value of oxygenated hemoglobin content in the negative group was significantly higher than that in the positive group. In the negative group, the situation difference was not significant (*p* = 0.085). In the positive group, the situation difference was not significant either (*p* = 0.689).

In view of the significant interaction between group and situation in channel 4, further simple effect test showed that there was no significant difference in the quality of adviser-advisee relationship between the two groups in thinking situation (*p* = 0.071), but there was significant difference in the group in simulated communication situation (F (1,40) = 8.259, *p* = 0.006 < 0.01, η2 = 0.171), the mean value of oxygenated hemoglobin in the positive group was significantly higher than that in the negative group. In the positive group, the situation difference was not significant (*p* = 0.064); In the low adviser-advisee relationship quality group, the situation difference was significant (F (1,40) = 10.030, *p* = 0.003 < 0.01, η2 = 0.200). The mean value of oxygenated hemoglobin content in thinking situation was significantly higher than that in simulated communication situation.

### Nirslab results

The preprocessed raw data were imported into nirslab software for a time series analysis, and the statistical parametric mapping (SPM) analysis with the Near-Infrared Spectroscopy-Statistical Parametric Mapping open-source software package (NIRS-SPM) was performed by referring to fMRI analysis method.

**Figure 2:**
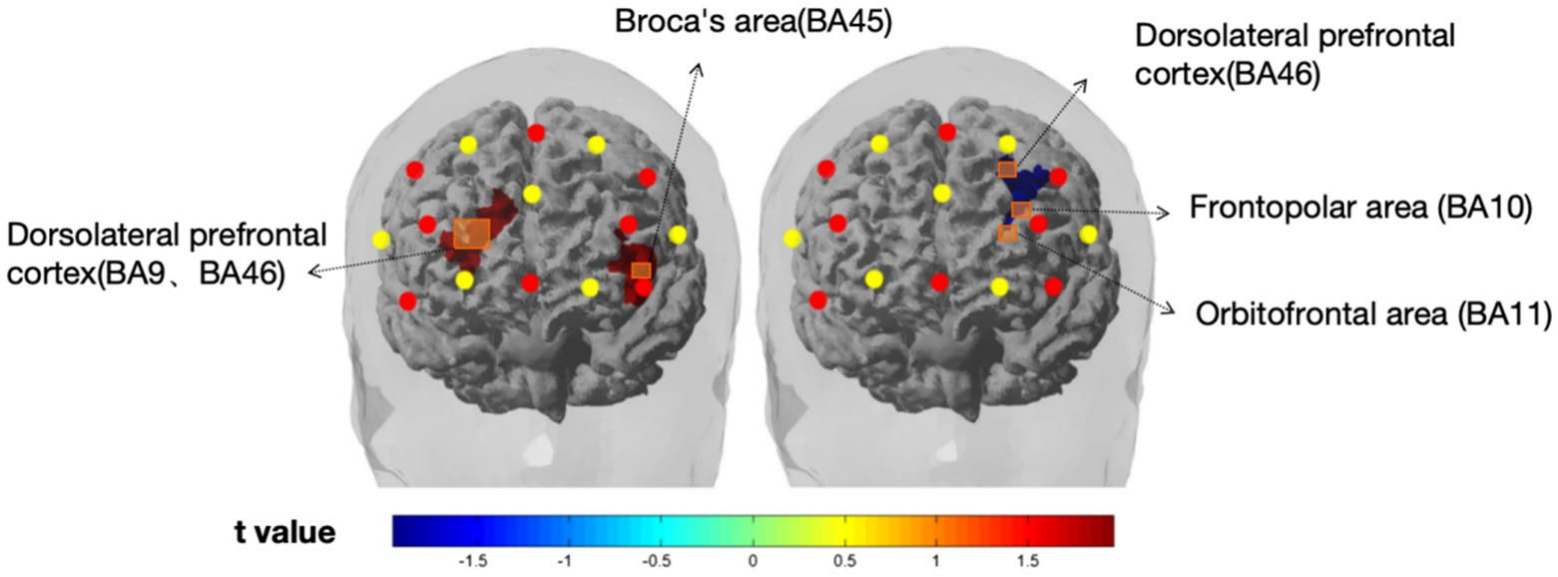
Comparison of brain activation between subjects in thinking situation (left) and simulated communication situation (right). Activation in the prefrontal cortex (DLPFC) during the t-test of the two groups in thinking situation (negative + positive) and simulated communication situation (negative + positive). Significant activations are displayed. *Note:* In the figure above, the subject faces forward, with the subject himself as the reference definition left/right, which is opposite to the reader’s perspective.

The main purpose was to combine the spatial location with the hemodynamic signal, view the activation region under each condition, and show the brain region. Then, the data of the participants processed by SPM were imported into nirslab software in the form of grouping, and the t-test was conducted on SPM level 2 module, and the significance *P* was set to 0.05. The results showed that the prefrontal regions of the two groups of subjects were significantly different under the conditions of thinking communication and simulation communication, and the specific differences in brain regions were shown in Fig. 2. Since different channels covering different specific Brodmann brain areas, the corresponding brain regions and their coverage for each significant channel are shown in Table 3.

**Table 3:** Brodmann areas corresponding to significant channels.

Channels	Brodmann areas	Coverage (%)
1	Broca's area(45)	70.6
	Dorsolateral prefrontal cortex (46)	29.3
2	Dorsolateral prefrontal cortex (9)	88.48
	Dorsolateral prefrontal cortex (46)	11.52
4	Frontopolar area (10)	38.26
	Orbitofrontal area (11)	42.13
	Dorsolateral prefrontal cortex (46)	6.96

The results of nirslab analysis showed that the activation of oxygenated hemoglobin in Broca's area (channel 1) and dorsolateral prefrontal cortex (channel 2) was significantly lower in the high adviser-advisee relationship quality group than in the low adviser-advisee relationship quality group under the thinking situation. However, in the simulated communication situation, the activation of oxygenated hemoglobin in the Frontopolar area (channel 4), orbitofrontal area (channel 4) and left dorsolateral prefrontal cortex (channel 4) in the positive group was significantly higher than that in the negative group.

## Discussion

Communication between advisers and students plays a vital role in students' academic progress and future significant development on the academic path. Undoubtedly, adviser-advisee communication and relationship are mutually constructed and influenced. Previous studies on communication between advisers and students have mostly focused on the behavioral level, leaving an explicit gap in directly understanding the communication between different adviser-advisee relationships at the neural level. To fill this gap, the present study employed a realistic presented problem task, based on which the HbO signals of participants with different adviser-advisee relationships were identified. The results of the fNIRS imaging technology supported our original hypotheses, suggesting that graduate students with better adviser-advisee relationship put less effort into thinking about how to communicate with advisers, but more effort into the actual communication act.

Specifically, first, the study found that during the thinking situation, the amount of oxygenated hemoglobin in both the Broca’s area (BA45) and dorsolateral prefrontal cortex (BA9, BA46) in the group with a positive adviser-advisee relationship was significantly lower compared to the group with negative adviser-advisee relationship. Essentially, the Broca’s area and dorsolateral prefrontal cortex were more easily activated during the thinking stage before communicating with the advisers in the group with less better adviser-advisee relationship.

Previous studies have found that the pars triangularis (a part of Broca’s area) is the main language motor area and is closely related to the production of language^29–31^. Following the research methods of EEG and functional brain imaging technology, Friederici proposed a three-stage theory of speech processing comprised of phrase structure, the processing of syntax and semantics, and the integration of different types of information^32,33^. The second stage of syntactic and semantic processing involves a series of brain regions in the frontal and temporal lobes—the BA44 area in the inferior frontal gyrus is mainly involved in syntactic processing, while the more anterior BA45/47 area is closely related to semantic processing^34^. This theory both describes the stage process of speech processing and explores the corresponding brain functional localization information^32,33^. This coincides with the current study’s results which stipulate that speech production and related brain areas (BA45) of the subjects were significantly activated in the process of communication. This also confirmed the effectiveness of the experimental operation from a side of brain. In our study, during the thinking process, students with worse adviser-advisee relationships are more likely to be activated in this area. One possible reason for this is that graduate students with negative adviser-advisee relationships were less willing to talk with their advisers, and may also have a kind of escape mentality for the adviser. Hence, in the face of the need to communicate with advisers and request his/her/their help to resolve the current dilemma situation, more mental preparation and cognitive resources must be mobilized to organize language, and complete semantic processing where the activation is more significant in Broca’s area.

Second, the results showed that in the simulated communication situation, the amount of oxygenated hemoglobin in the Frontopolar area (BA10), orbitofrontal area (BA11), and left dorsolateral prefrontal cortex (BA46) of the positive adviser-advisee relationship group was significantly higher than that of the negative relationship group. The abovementioned brain region of the positive relationship group were more easily activated than those of the negative group, with the difference being concentrated in the left side. Participants with positive adviser-advisee relationship were likelier to be activated in the frontal pole area (BA10) in the simulated communication situation, and there may be two possible reasons for this result. Firstly, our finding coincided with previous studies on the frontal pole area and prospective memory. Many studies have confirmed the involvement of the frontopolar area in the regulation of prospective memory by neuroimaging techniques ^35,36^. One study even demonstrated that transient suppression of Brodman’s area 10 (Frontopolar area) by transcranial magnetic technology (TMS) impaired prospective verbal memory^37^. Accordingly, Costa^38^designed a TMS intervention experiment to explore the role of frontal polar cortex in visuospatial prospective memory, and the results also proved that frontal polar area plays a key role in the prospective memory process. Although asymmetric neural activity patterns associated with prospective memory related tasks have been demonstrated by functional neuroimaging techniques^39^, these are characterized by stronger activation in the left frontal lobe than in the right which also coincides with the results of this experiment,when communicating, only the activation of the left Frontopolar area in the group with positive adviser-advisee relationship was significantly higher than that in the other group. Secondly, some scholars have explored the functional characteristics of the Frontopolar area in cognitive activities and found that the frontal polar cortex is also related to opinion selection and theory of mind^40–43^. Research on interpersonal communication and interaction has likewise shown that the ability to choose ideas and theory of mind is the key to good communication and successful relationship building^44–47^. Frontopolar areas play an important role in theory of mind processing. This experiment simulated the situation of communication with advisers. When thinking about communicating with the advisers to solve the current dilemma, the individual may also use the ability of theory of mind to imagine the adviser’s expected response to fully organize the effective communication content that is likelier to garner his/her/their help. The activation of the Frontopolar area was more significant in the group with positive adviser-advisee relationship: this may be due to the good use of opinion selection and theory of mind to initiate effective communication and build a positive adviser-advisee relationship. What’s more, there also exits studies having shown that the involvement of the left and right frontal lobes depends on the integration and complexity of the task, respectively^48,49^. However, no studies have yet directly revealed significant hemispheric differences in frontal polar brain activation^50^.

The current study also found that participants with better adviser-advisee relationships were more likely to be activated in the orbitofrontal region during simulated communication scenarios, and this result is likely related to the reward circuit. The reward system is a specific circuit in the human brain that processes reward information, including the orbitofrontal lobe, cingulate gyrus, amygdala, subcortical striatum, insula, etc^51,52^. It is composed of the main migratory brain regions of dopamine—an important neurotransmitter for the brain to complete the representation of reward information^53^. Further studies showed significant differences in the specific division of brain regions in the reward system during the execution of reward-related cognitive functions, and the orbitofrontal area was mainly involved in the value representation of reward information^54,55^. Some posit that the activation intensity of the orbitofrontal area is related to the expected or actual reward intensity and value^56–58^. In the current study, subjects with better adviser-advisee relationship showed more significant activation in the orbitofrontal area than those with wores adviser-advisee relationship. This may be due to the fact that postgraduate students with better adviser-advisee relationship expect higher expected benefits from adviser’s help to solve their current predicament. Correspondingly, the subjects in the group with negative adviser-advisee relationship may not have high expectations for getting out of the current predicament with the communication, hence the reward intensity expected in their cognition is also lower than that of the subjects with positive adviser-advisee relationship.

Finally, there were also differences between two groups in the activation of the Dorsolateral prefrontal cortex during both the thinking and the simulated phases.The study of Dorsolateral prefrontal cortex (DLPFC) has been a salient topic in the field of cognitive cortex studies. Many have found that the dorsolateral prefrontal cortex is an important brain area in the executive control network of the brain, which is mainly responsible for cognitive monitoring and inhibition^59,60^. When thinking about how to communicate with the adviser, the dorsolateral prefrontal cortex of the postgraduate students in the negative adviser-advisee relationship group was significantly activated compared with the other group, which may just reflect the involvement of executive control function in the process of thinking about how to communicate with the adviser. Furthermore, the conflict monitoring theory^61^ proposed by Carter and Botvinick may also explain the situation. The theory suggests that a conflict-detection module in the anterioringulate cortex (ACC) monitors conflict and signaling when individuals are in highly conflicting situations^62,63^. Later research results also provided evidence to the validity of the theory. For example, previous meta-analyses have found that the dorsolateral prefrontal cortex is essential for the involvement of inhibitory control^64,65^, and it particularly performs the function of cognitive conflict monitoring in the context of high conflict^64^. After receiving the signal, the dorsolateral prefrontal cortex (DLPFC), as a conflict control module, integrates individual cognitive resources and inhibits the processing of interference stimuli. This makes individuals concentrate more cognitive resources on core goals to effectively resolve conflicts^66,67^. Simultaneously, studies have also shown that DLPFC also plays an important in executive function^68^, working memory^69^ and emotion regulation^70,71^. Following the results of these previous studies, it is reasonable to speculate that in the current study, the graduate students in the negative relationship group may be in a highly conflictive situation– they will therefore not choose to communicate with the adviser for help when encountering difficulties in daily learning and life. In the experiment task on thinking about communicating with the adviser to solve the current dilemma, the person must suppress it or confront the contradiction between high conflict situation cognition, suppress the real reaction, and confront the situation in contrast to their usual behavior of response, while also having higher quality than the learning relationship group, which was more tense and experienced cognitive conflict. This process ultimately leads to significant DLPFC activation. Additionally, this behavior may also involve many other components of executive function, such as working memory, executive function, and emotion regulation, which may also enhance the activation of DLPFC. While in the same brain region of the dorsolateral prefrontal cortex (DLPFC), the group with positive adviser-advisee relationship showed a higher level of activation in the simulated communication situation, with the difference being only shown in the left dorsolateral prefrontal cortex. On the one hand, this may be related to hemispheric differences in inhibitory function of the brain. A meta-analysis of the neural mechanisms controlling inhibition in the brain has shown that different types of inhibitory responses such as cognitive inhibition, emotional inhibition, and reflective inhibition are involved in different neural tissues^72^. However, regardless of the type of inhibitory control process, the brain regions responsible for activation are mainly in the right hemisphere^73–75^. On the other hand, this may also be related to the functional differences between the left and right sides of the dorsolateral prefrontal cortex. Some scholars used transcranial direct current stimulation (tDCS) system to stimulate DLPFC on both sides of the subjects where they found that DLPFC showed brain lateralization when suppressing interfering information related to emotion. Specifically, the right DLPFC was significantly activated when suppressing negative interference information^76,77^. The left DLPFC plays a more important role in suppressing positive interference information^78,79^. Tomarken et al^80^*.* also proposed that the top-down processing of emotion regulation is mainly controlled by the dorsolateral prefrontal cortex^80–83^. Moreover, neuroimaging studies have shown that DLPFC's response to emotional stimuli is asymmetric—that is, in the face of negative emotional valence and aversive stimuli, the right DLPFC of the participants is preferentially activated. The left DLPFC meanwhile is preferentially activated in response to stimuli with positive emotional valence i.e., stimuli that are attractive in nature^84^. In the current study’s simulated communication situation, the left dorsolateral prefrontal cortex of the subjects in the positive adviser-advisee relationship group was more significantly activated, indicating that the process of communicating with the adviser on the current difficulties and asking for help was a positive valence stimulus for master students with better adviser-advisee relationship, because the left DLPFC was significantly activated. This also confirmed the previous research on cerebral inhibitory function and dorsolateral prefrontal function hemispheric differences.

## Conclusion

By adopting the task paradigm of realistic presented problem, the current study used the functional near-infrared spectroscopy techniques to explore the distinct brain mechanism of graduates with different advider-advisee relationships when thinking and stimulating a conversation with their advisers. The results showed significant interactions between the two groups in channels 1, 2 and 4. Specifically, the Broca’s areas (channel 1) and dorsolateral prefrontal cortices (channel 2) were more easily activated in the pre-communication thinking situation of postgraduate students with negative adviser-advisee relationship. Moreover, in the simulated communication situation, the activation of brain regions in frontal pole area (channel 4), orbitofrontal area (channel 4), and the left dorsolateral prefrontal cortex(channel 4) was more significant in postgraduate students with positive of adviser-advisee relationship. The altered brain activation of graduates during the task may be relevant to the two groups’ differences in verbal expression ability (Broca’s triangle), opinion selection and theory of mind (frontal polar region), expectation and emotional value of communication with advisers (orbital frontal region), and emotional valence and cognitive conflict in the face of communication with advisers (dorsolateral prefrontal region). All in all, our study found the characteristics of cognitive neural network of postgraduate students with different adviser-advisee relationships when communicating with their advisers, thus providing an empirical research basis for further exploring the construction of better adviser-advisee relationships.

## Data Availability

The datasets used and/or analysed during the current study available from the corresponding author on reasonable request.
